# Computational development of mushroom-6-glucan/paclitaxel as a synergistic complementary medicine for breast cancer therapy

**DOI:** 10.1186/s12906-025-04772-7

**Published:** 2025-02-15

**Authors:** Nehal M. EL-Deeb, Omar M. Ibrahim, Ayman M. Kamel, Ahmed I. Gomaa, Ahmed M. Kenawy

**Affiliations:** 1 Pharmaceutical Bioproducts Research Department, Genetic Engineering and Biotechnology Research Institute (GEBRI), City of Scientific Research and Technological Applications (SRTA-City), New Borg El- Arab City, Alexandria Egypt; 2 Department of Medicine and McDonnell Genome Institute, Washington University School of Medicine, St. Louis, MO 63110 USA; 3 Pharmaceutical Sciences Division, Medicinal and Aromatic Plants Department, National Research Centre, Cairo, Egypt; 4 School of Nutrition Sciences, Faculty of Health Sciences, University of Ottawa, Ottawa, ON Canada; 5 National Research Center, Nutrition and Food Science Department, Doki, Giza Egypt; 6 Nucleic Acids Research Department, Genetic Engineering and Biotechnology Research Institute, City of Scientific Research and Technological Applications (SRTA-City), New Borg El-Arab City, Alexandria Egypt

**Keywords:** 6-glucans, Polysaccharides, Chemoresistance, Paclitaxel, Cancer targeting

## Abstract

**Background:**

Breast cancer is chemo-resistant and highly metastatic, often resulting in patient mortality. One of the primary factors contributing to the metastasis and chemotherapy resistance is the presence of cancer stem-like cells. We posited that the natural polysaccharide known as 6-glucans, derived from *Pleurotus ostreatus*, could effectively counteract the chemotherapy resistance associated with cancer stem-like cells in breast cancer.

**Methods:**

We computationally developed a specific dual combinatorial therapy involving 6-glucans and Paclitaxel (PTX) and tested on preclinical 3D mammosphere human tumor models representing receptor-positive and receptor-negative breast cancer. Using this preclinical 3D spheroid technology, we tested the anti-cancer properties of these predicted treatment combinations on mammospheres containing human breast cancer stem cells.

**Results:**

Among the 40 distinct combinations examined, computational prediction revealed that the addition of 2.0 mg/mL of 6-glucans to a low dose of 3.0 µg/mL PTX was the sole combination demonstrating a synergistic effect. This optimized synergistic combination therapy displayed a significant inhibitory impact on human cancer epithelial and stem cell migration, evasion, and colony formation. The inclusion of 6-glucans also augmented apoptosis in both breast cancer cells and stem cells, leading to a six-fold reduction in BrdU labeled cells and an increased arrest of cells in the sub-G0 phase. These effects were mediated through mitochondrial dysfunction and the downregulation of associated oncogenes.

**Conclusion:**

Our study revealed that the computationally predicted 6-glucans-based binary complementary medicine exhibited sequence- and concentration-dependent anticancer synergistic effects.

**Supplementary Information:**

The online version contains supplementary material available at 10.1186/s12906-025-04772-7.

## Introduction

Breast cancer is the most frequently diagnosed malignancy and the leading cause of cancer-related mortality among women worldwide [1, 2]. Despite the reported treatment strategies against breast cancer were such as surgical resection, adjuvant chemotherapy, radiotherapy, and hormone therapy, the efficacy of these strategies remains an unsatisfactory solution for this problem. This reduced efficacy is attributed not only to the increasing drug resistance, but also to the limitations in applying certain therapeutic strategies in some types of cancer. The accumulation of causes and consequences of cancer further complicates efforts to address it effectively [3–6]. For example, triple-negative breast cancer, the most lethal breast cancer subtype, can only be treated by cytotoxic chemotherapy and does not respond to the hormonal or trastuzumab-based therapies [7]. Breast cancer can lead to paraneoplastic syndromes (PNS), which are rare disorders triggered by the body’s immune response to cancer. These can cause neurological symptoms and affect various body systems. Chemotherapy, a common treatment for breast cancer, can also cause side effects such as cognitive issues, fatigue, neuropathy, and a weakened immune system. Long-term effects can include emotional distress, heart problems, and joint pain. Both breast cancer and its treatment can lead to a range of debilitating effects that can significantly impact a patient’s quality of life [8]. Unfortunately, some patients can develop resistance to the available chemotherapeutic treatments and usually die due to metastasis [9–11]. There is a strong evidence that breast cancer stem cells drive metastases and are the main reason behind chemotherapy standard of care drug resistance in breast cancer patients [12]. Recently, combination therapy using traditional drugs along with natural agents or synthetic drugs that affect significant pathways in cancer cells was reported to offer a means of competency for overcoming cancer Multiple Drug Resistance (MDR) and reducing chemotherapy side effects [13–22].

Paclitaxel is a natural plant alkaloid isolated from the bark of the pacific yew trees. The active compound was firstly isolated and named as Taxol by Schwab et al. [23–25]. Paclitaxel was successfully used in the treatment of various cancer types (cervical, breast, ovarian, brain, bladder, prostate, liver, and lung cancers) as an excellent chemotherapy strategy, which is the most common and cost-effective treatments for controlling breast cancer. Nevertheless, clinical applications of Paclitaxel are significantly limited due to the development of MDR [26–36]. Hence, a continued hard work is needed for improving the current regimen’s efficacy and overcoming their adverse drawbacks.

Polysaccharides extracted from mushrooms are known to have both direct and indirect antitumor activities on various allogeneic and syngeneic tumors and showed the capability to prevent tumor metastasis. β-(1,3)/(1,6) D-glucans are long chain polymers of natural glucose produced by mushroom cell walls, which have been shown to have numerous immunomodulatory properties [33]. We have previously purified 6-glucans polysaccharides from *Pleurotus ostreatus* and confirmed its ability to induce natural killer cell’s (NK-cell) cytotoxicity against several cancer types with a higher potency specifically against breast cancer [37]. Despite the known immunomodulatory activity of 6-glucans, its direct antitumor activity is remaining unknown. Accordingly, we sought to investigate the potential synergistic effects that may result upon combining 6-glucans with Paclitaxel as an anticancer treatment to overcome the development of MDR towards Paclitaxel chemotherapy. The importance of preclinical 3D spheroid technology in studying cancer behavior lies in their ability to replicate key characteristics of human solid tumors. By studying tumor cells in 3D spheroids, we can gain insights into the molecular mechanisms underlying invasion, and colonization, which are critical steps in tumor behavior [38]. Here we hypothesized that the natural 6-glucan mushroom polysaccharide will overcome chemotherapy resistance in breast cancer.

## Methods

### Materials

All cell lines were purchased from American Type Culture Collection (ATCC). Human breast cancer MCF-7 cells (ATCC HTB-22), mammary gland/breast adenocarcinoma MDA-MB-231cells (ATCC-HTB-26^Tm^), and mammary gland/breast adenocarcinoma SK-BR-3 (ATCC^®^- HTB-30) were cultured in RPMI media (Sigma) supplemented with 10.0% FBS (Lonza), 4.0 mM L-glutamine, 1.0 mM sodium pyruvate ((all from Sigma), 1.0% penicillin/streptomycin (Lonza).

*Pleurotus ostreatus* (Jacq.) P. Kumm. (type NRRL-0366), NCBI: txid5322, was provided by the Agricultural Research Service (Peoria, U.S.A.), the mushroom spawns were prepared in order to cultivated mushroom as described in our previous work (El-Deeb et al. 2019). 6-glucans polysaccharides were extracted from the fruiting bodies of the cultivated *P. ostreatus*, 6-glucans polysaccharides were purified and identified as described in our previous work (El-Deeb et al. 2019).

### Cell viability/toxicity assays

All cell lines were purchased from the American Type Culture Collection (ATCC). Human breast cancer MCF-7 cells, mammary gland/breast adenocarcinoma MDA-MB-231 cells, and mammary gland/breast adenocarcinoma SK-BR-3. Cells were incubated at 37 °C in 5.0% CO2. The semi-confluent layers of each cell line were treated with PTX or 6-glucans different concentrations for 24,48 and 72 h. Cell viability/toxicity was measured using two corresponding tests (i) CellTiter Aqueous One Solution -MTS- assay (Cell proliferation), which quantifies the reduction of tetrazolium compound into soluble formazan by dehydrogenase enzymes existing in living cells. (ii) ATP Cell Viability Luciferase Assay (Promega) as previously described [39]. Cellular viability was calculated using standard curve, which was prepared using two-fold serial dilutions of cells in a 96-well plate using RPMI media and the assays were performed as previously described. Luminescence was measured 10 min after reagent addition, using a GloMax^®^-Multi + Detection System [40].

### Isolation of CD44+/CD24 − breast cancer stem cells (CSCs)

MCF-7 and MDA-MB-231 cells were grown in RPMI media with 10% FBS until sub-confluence. Cells were collected from the tissue culture flasks via trypsinization then centrifugation. The collected cells were washed with PBS (pH 7.4), MCF-7 cell pellet was stained with APC-conjugated CD44 antibody (BD Pharmingen, USA) and prep cy5-conjugated CD24 antibody (BioLegend, USA). While, MDA-MB-231 cells pellet was stained with APC-conjugated CD44 antibody, prep cy5-conjugated CD24 antibody, and Alexa-fluor ALDH antibody (BioLegend, USA). After incubation with the antibodies, the excess antibodies were washed off using PBS and the cells were sorted by a fluorescence-activated cell-sorting device (FACS Aria II: BD P469000021).

### Inhibition of the sorted CSCs mammospheres formations and cell viability assays

For the formation of breast cancer spheroids, the hanging drop assay was used. Briefly, MCF7 and MDA-MB-231 sorted CSCs cells were adjusted to the count of 50 cells/20.0 µl in RPMI media (with or without the treatment: PTX, 6-glucans and the selected combination). Once the mammospheres were formed, they were transferred into ultra-low attachment 96-well plates for performing the cytotoxicity assays of PTX, 6-glucans, and their combinations, as described above, using MTS and ATP assays.

### Synergism and antagonism between paclitaxel and 6-glucans combinations

6-glucans polysaccharide was isolated and purified as described in our previously research article [37]. Forty different combinations of PTX and 6-glucans were assessed to identify any possible synergistic interactions to develop a novel treatment strategy. So that, we constructed different combinations between Paclitaxel and 6-glucans as a synergy prediction pipeline using Combenefit software [41]. Combenefit software enables model-based quantification of drug combinations by comparing additive and actual effects for given dose-response data. The obtained results of MTs and ATP cytotoxicity assays of both 2-D and the sorted CSCs mammospheres after treating them with the individual or combined doses (6-glucans: starting from 0.25 to 2.00 mg/ml and PTX: from 0.16 to 3.00 µg/ml). The data were processed using Classical Synergy Models, Loewe, as a single experiment or in batches for high throughput screening.

### Colony forming assay of the CSCs sorted cells

The effects of different treatments of paclitaxel, 6-glucans, and the selected combination on the ability of the sorted CSCs cells to form colonies were assessed. Briefly, CSCs sorted MCF7 cells were plated in 0.4% agarose (with or without the treatment: PTX, 6-glucans or the selected combination) with a 1.0% agarose underlay (4 × 10^4^ cells per well in six-well plates). Then, the cells were incubated for 14 days. The culture media (with or without the treatment: paclitaxel, 6-glucans, or the selected combination) were changed every 2 days. Colony formation was expressed as a percentage (relative to the control, untreated cells). Finally, clonogenic assay was performed in triplicate.

### Trans-well migration assay

The effects of treatments on the migration ability of the sorted CSCs cells were assessed using Trans-well migration assay. Matrigel (BD Biosciences: stored at − 20 °C) was thawed at 4.0 °C overnight, the Matrigel was diluted in a serum-free medium. Aliquot of 30.0 µl diluted Matrigel was transferred to the upper chamber to form a gel at 37 °C. The sorted MCF7 and MDA-MB-231 CSCs cells were grown to 80% confluence in complete RPMI media, then, cells were starved overnight before experiment. The collected cells were washed in PBS (pH 6.8) and incubated in 24-well BD Biosciences Trans-well Chambers (BD BioCoat Control inserts equipped with 8.0-µm pore-size polycarbonate membrane). The collected cells were seeded at 5 × 10^4^ cell/well in 0.5 ml RPMI media (with or without one of the treatments: PTX, 6-glucan, and the selected combination) in the upper well. The lower wells were filled with 600 µl of media. After 24 h incubation, the cells were wiped from the well with a cotton swab, fixed with 4.0% paraformaldehyde, and stained in 10.0% crystal violet. The migrating cells were counted, and images were captured using Olympus microscope (Olympus, Tokyo, Japan). All experiments were performed in triplicates and nine fields were counted per filter in each group.

### Migration assay

The sorted MCF7 and MDA-MB-231 CSCs cells were grown to 95.0% confluency in a complete RPMI medium and then wounds were produced using a plastic pipette tip or scraper. After washing with a pre-warmed phosphate buffer saline (PBS), the plates were incubated with complete RPMI medium or the treatment: PTX, 6-glucans or the selected combination of both treatments. After 24 h of incubation, the cells were washed with PBS and migration efficacy was examined under light microscope (Olympus, Tokyo, Japan). Wound width was measured for both treated and untreated groups. The migration assay was performed in triplicate.

### Annexin V/propidium iodide assay for apoptosis evaluation using flowcytometry

The effect of selected combination to induce cellular apoptosis and necrosis on both 2-D and spheroids of the sorted MCF7 and MDA-MB-231 CSCs cells was assessed using flow cytometry and annexin V-FITC apoptosis detection kit, according to the manufacturer’s instructions. This assay can distinguish between apoptosis and necrosis, when staining annexin V-FITC with PI staining (PI detect necrotic cells with permeabilized plasma membrane). Briefly, cells were treated as described above, incubated for 24 h. Then, cells were resuspended in 500 µl of 1X binding buffer and stained for 15 min in the dark using 5 µl of annexin V-FITC and 5 µl of propidium iodide. After incubation, the cells were analyzed by a BD Falcon flow cytometer (BD Falcon, USA).

### BrdU proliferation assay

For the quantification of 5-bromo-2-deoxyuridine (BrdU) incorporation into DNA, both 2-D and spheroids of the sorted MCF7 and MDA-MB-231 CSCs cells were plated at a seeding concentration of 4 × 10^5^ cells/ml in T25 flasks and ultra-low attached 24 plates for 24 h. After incubation, BrdU (BD PharmingenTM BrdU Flow Kits) was added to the culture medium at a final concentration of 10 µM and incubated for another 24 h. Total DNA was collected and stained with DAPI for cell cycle analysis.

### Measurement of mitochondrial membrane potential and the induced intracellular reactive oxygen species (ROS)

ΔψM is an important parameter of mitochondrial function that has been used as an indicator of cell health. Variations in ΔψM were previously studied using cationic dyes, such as rhodamine-123 (Rh123) and DiOC6. In healthy cells with high ΔψM, JC-1 forms complexes known as J-aggregates with intense green fluorescence. However, in cells with low ΔψM, JC-1 remains in the monomeric form, which exhibits a red fluorescence. The higher the ratio of red to green fluorescence is, the higher the polarization of the mitochondrial membrane. So that, Cayman’s JC-1 Mitochondrial Membrane Potential Assay Kit was used to study the effect of paclitaxel/6-glucans treatment combinations on mitochondrial behavior of the sorted MCF7 and MDA-MB-231 CSCs mammospheres. Changes in ΔψM reflected by different forms of JC-1 as either green or red fluorescence were determined as a ratio of green: red using confocal microscopy technique. Cells were treated with 100 µl of JC-1 staining solution per ml of culture medium for 15–30 min, then the cells were analyzed directly using Leica SP5 Scanning Confocal Microscope (Leica, Japan).

Cells were plated in 24-well plates with concentration 5 × 10^4^ cells/well, and then treated as described previously. After incubation, the cells were stained by 2’,7’ –dichlorofluorescin diacetate (DCFH-DA, 10 µM) for 20 min at 37 °C in the dark. After incubation, the fluorescent intensity of the formed.

DCF was photographed using a fluorescence microscope (Olympus, Tokyo, Japan) and detected by fluorescence spectroscopy with excitation / emission at 485 nm / 535 nm. The ROS Inducer (Pyocyanin) was used as a positive control at final concentration 10 nmol.

### Effect of paclitaxel/6-glucans treatment combinations on different apoptotic gene expression

Gene expression level of *TGF*, *Survivin*, *BCL2*, and *MMP7* genes were analyzed in both PTX/6-glucan treated and untreated (control) sorted MDA-MB-231 CSCs mammospheres using two steps RT-qPCR technique (Light Cycler fluorimeter Bio-Rad S1000 Tm thermal cycler (Bio-Rad, USA). RNA extraction was performed using TRIzol-based RNA extraction kit according to the manufacturer instructions. cDNA was then synthesized via Thermo Scientific cDNA Synthesis Kit using 10 µl of the obtained RNA according to the manufacturer’s instructions. RT-qPCR reactions were performed in triplicates, where 1 µl of the cDNA was incorporated with Maxima SYBR Green Master Mix and 5.0 pmol of each forward and reverse gene specific primers for each sample. The PCR cycle program was done in a BioRad Real-Time PCR (BioRad, USA) using the following cycling program: 1cycle 95 C for 1 min followed by 40 cycles of 15 s at 94 °C, 30 s at the appropriate annealing temperature for each pair of primers, and 30s at 72 °C. The Cq values were normalized using the β-actine reference gene, then the mean relative gene expression levels and fold expression were analyzed by CFX manager Software version 3.0.1 (BioRad, USA).

### Statistical analysis

Statistical analyses and calculations of IC_50_ values were performed using Graph Pad Prism V.9.0. All statistical tests were performed at *p* values ≤ 0.05 using one- and two-way analysis of variance (ANOVA). The numbers presented in the text indicate the mean values of three replicates of triplicated experiments. The data were represented as mean ± standard deviation (SD) for bio-distribution studies and means ± standard error of the mean (SEM) for other studies.

## Results

### 6-glucans anticancer effects against breast cancer cell lines

**Fig. 1:**
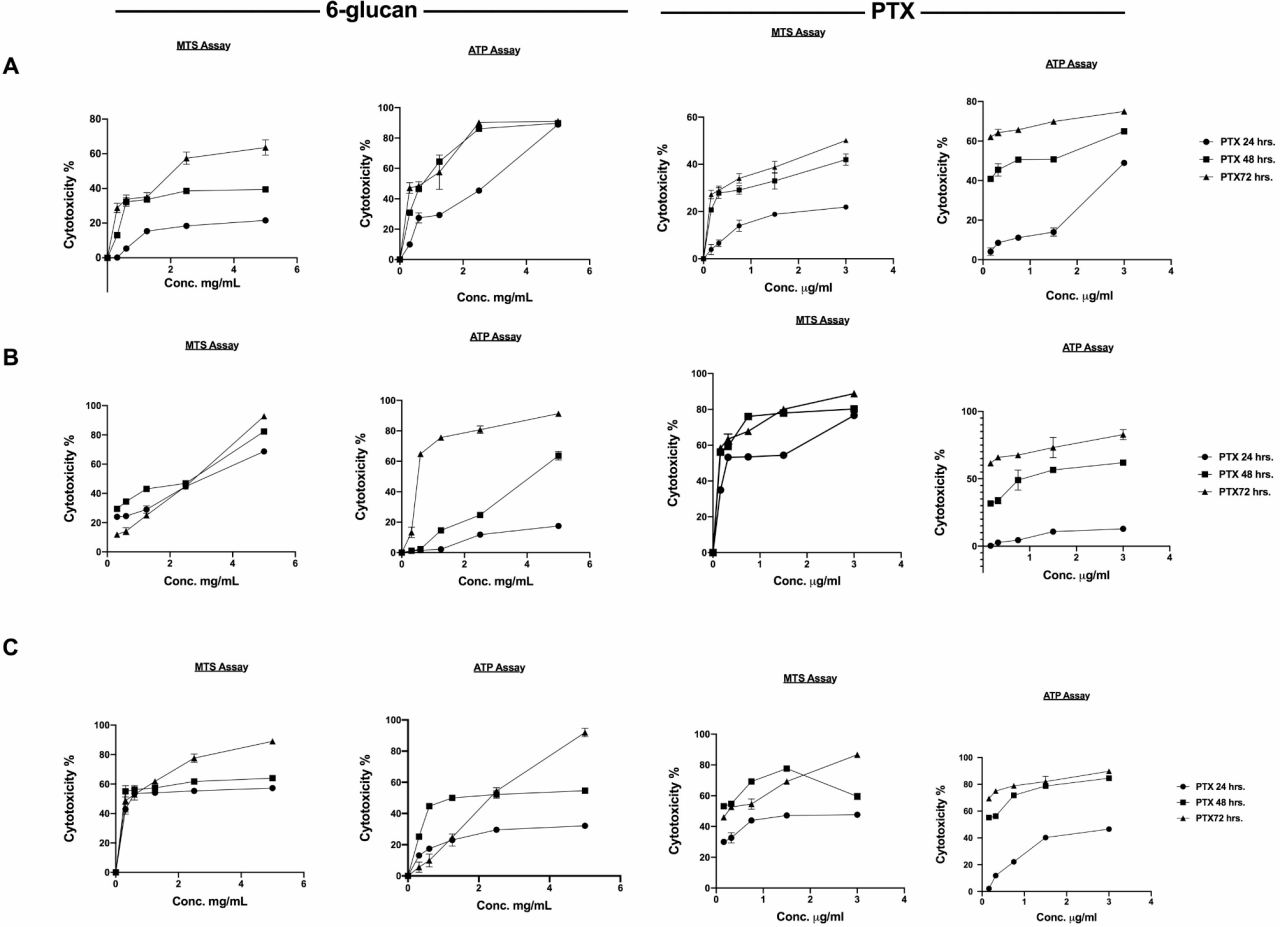
Anticancer Effects of 6-Glucans and PTX on Breast Cancer Cell Lines. A-C illustrates the antiproliferative effects of 6-glucans and PTX on hormone receptor-positive and highly aggressive metastatic receptor-negative breast cancer cell lines, including MCF-7, SK-BR-3, and MDA-MB-231. Using a MTS-based and ATP assays, the graphs display the response of different cell lines to various concentrations of 6-glucans (0.03 to 5.00 mg/ml) and PTX (0.16 to 3.00 µg/ml) over treatment periods of 24, 48, and 72 h. The results demonstrate the dose-dependent cytotoxicity of the treatments across different cell lines and time points

**Fig. 2:**
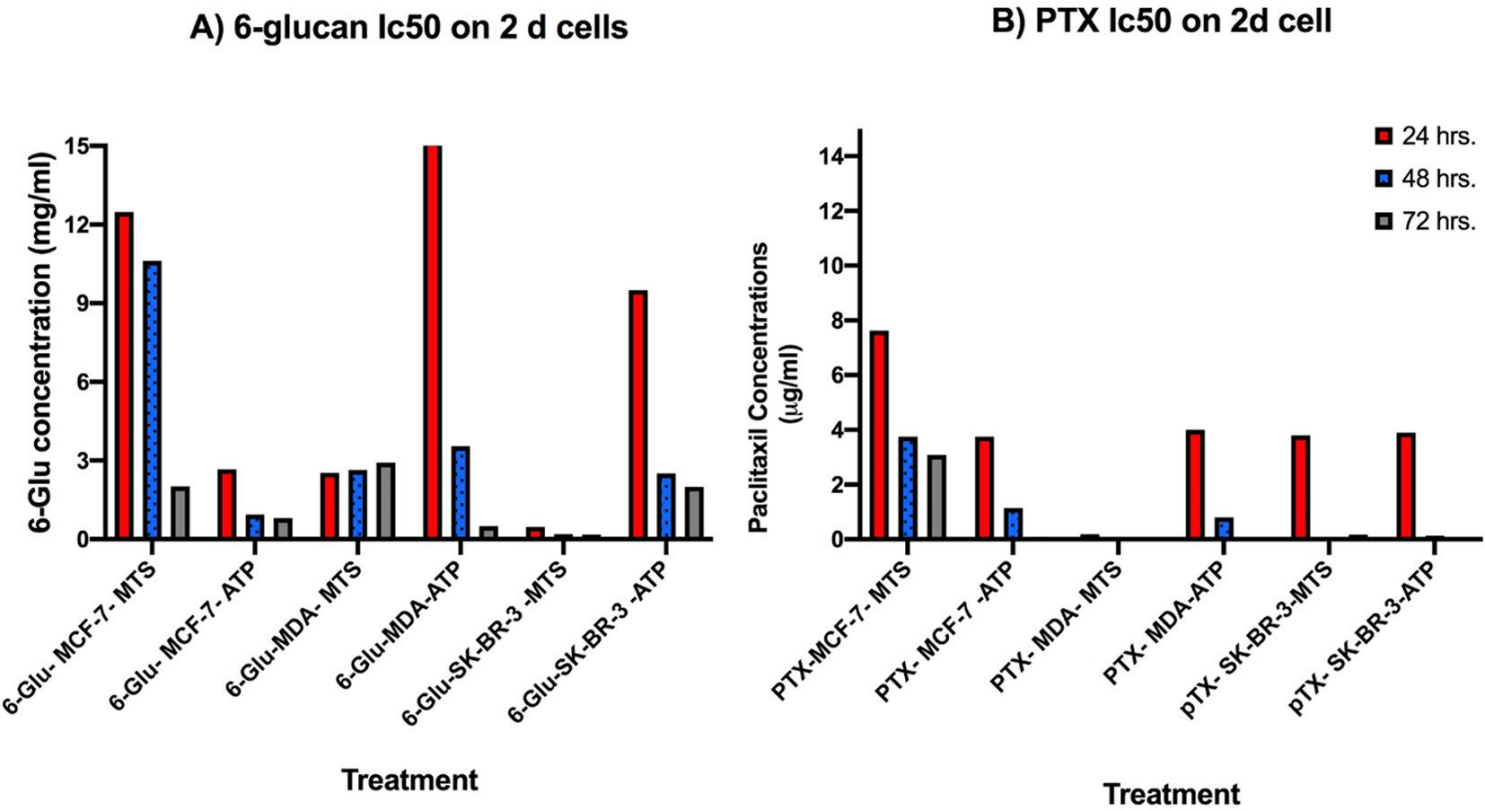
IC_50_ values of 6-glucans and PTX on 2-D breast cancer cell lines. The IC_50_ values were calculated after treatments of 2-D breast cancer cell lines with different 6-glucans concentrations (0.03, 0.06, 1.25, 2.50, and 5.00, mg/ml) and PTX concentrations (0.16, 0.32, 0.75, 1.50, and 3.00 µg/ml) for 24, 48, and 72 h

In order to investigate the direct anti-tumor effect of 6-glucans against breast cancer, cell proliferation MTS based assay and cellular ATP assay were used. The anti-proliferative/cytotoxic effect of 6-glucans and PTX against the receptor positive and the highly aggressive/metastatic receptor negative breast cancer cell lines: MCF-7, SK-BR-3, and MDA-MB-231 cell lines, (Fig. 1A-C) was screened. All cell lines were treated with 6-glucans at concentrations of 0.03, 0.06, 1.25, 2.50, and 5.00, mg/ml and PTX at concentrations 0.16, 0.32, 0.75, 1.50, and 3.00 µg/ml for 24, 48, and 72 h. Upon cellular treatment, all cell lines showed distinct patterns in their sensitivity to both PTX and 6-glucans. Both SK-BR-3 and MDA-MB-231 cell lines were more susceptible to the tested compounds, when they were compared with MCF-7 cells. Upon 6-glucans treatment, the rates of proliferation of SK-BR-3 and MDA-MB-231 cells were declined to 35.4 and 18.2%, respectively after 48 h. In addition, 53.44% and 61.64% reduction in cellular ATP was recorded after treating cells for 48 h with 5.0 mg/ml 6-glucans (Fig. 1A-B). These proliferation percentages were further dropped to 9.5% and 7.5%, respectively, after 72 h incubation with further reduction in cellular ATP (93.8% and 90.92%, respectively). In comparison to SK-BR-3 and MDA-MB-231, MCF-7 was more stable against the treatment and both 6-glucans and PTX were less cytotoxic to the cells (proliferation rate reached 39.45 and 50.17, respectively). Meanwhile, the reduction percentages of cellular ATP were 90.90 and 74.5% at low concentrations of 6-glucans and PTX, respectively. Furthermore, using MTS assay, the calculated IC_50_ values after 24 h of treatment using 6-glucans and PTX on MCF-7 cell line reached 12.48 mg/ml (Fig. 2A) and 9.6 µg/ml for both 6-glucans and PTX, respectively (Fig. 2B). These IC_50_ values dramatically dropped after 72 h of treatments with 6-glucans and PTX (Fig. 2B). However, 6-glucans and PTX IC_50_ values after 48 h of treatment on MDA-MB-231 were 2.9 mg/ml and 0.21 µg/ml, respectively. Furthermore, the lowest IC_50_ values of 6-glucans and PTX were recorded after treating SK-BR-3 cell line for 48 h, the values were 0.18 mg/ml and 0.1699 µg/ml, respectively. Therefore, the combination of 6-glucans and PTX significantly reduced cellular ATP in cancer cells. The effectiveness of the treatment, measured by the IC50 value, improved over time, with the most significant drop after 72 h of treatment. The lowest IC50 values were recorded when treating the SK-BR-3 cell line for 48 h, indicating the highest effectiveness of the treatment combination.

### Anticancer activity of 6-glucans on mammospheres formation of the sorted CD44+/CD24−/low breast cancer stem cells (CSCs)

**Fig. 3:**
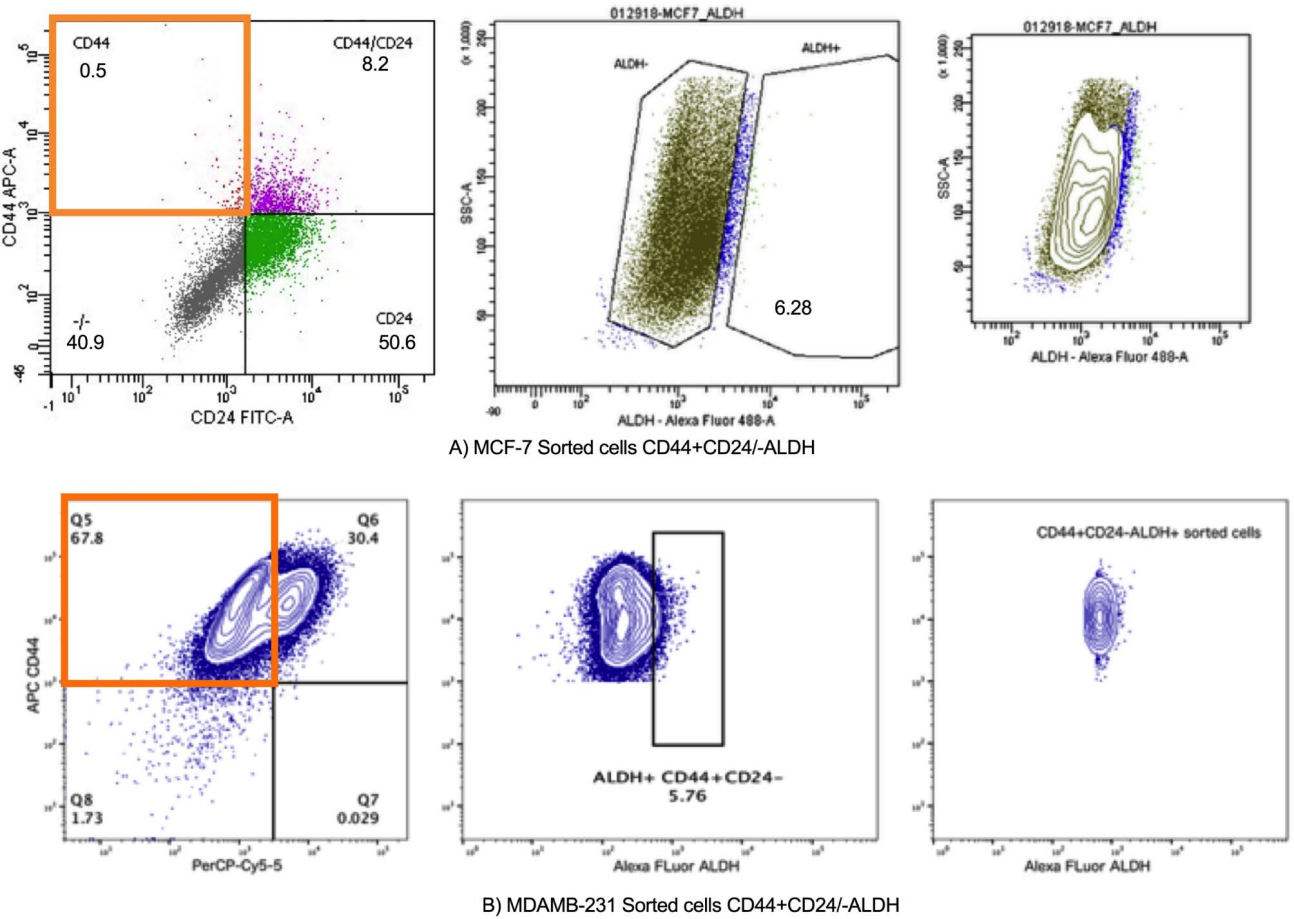
Detection of ALDH+/CD44+/CD24 − Stem cell-like Populations in Breast Cancer Cell Lines. **A**. shows the gating strategy for identifying the ALDH+/CD44+/CD24 − populations in MCF-7 cells. **B**. displays the corresponding sorting in MDA-MB-231 cells. The cells were labeled with antibodies against ALDH, CD44, and CD24 and then sorted using a FACS Aria II instrument, with the positively selected populations highlighted in the flow cytometry plots

To examine the stem features of the different breast cancer cell lines, we assessed the expression of CD44, CD24 and ALDH1 in both MCF-7 and MDA-MB-231 cell lines using FACS. Cells that are CD44 positive and CD24 negative or low are generally considered to have stem cell-like properties in breast cancer. FACS analysis revealed the expression of ALDH, CD44 and CD24 in both cell lines and as expected, MDA-MB-231 cell lines exhibited higher CD44+/CD24 − feature than MCF-7 cell line (Fig. 3). while MCF-7 cell line showed higher CD24+/CD44- feature than MDA-MB-231 cell lines (Fig. 3).

**Fig. 4:**
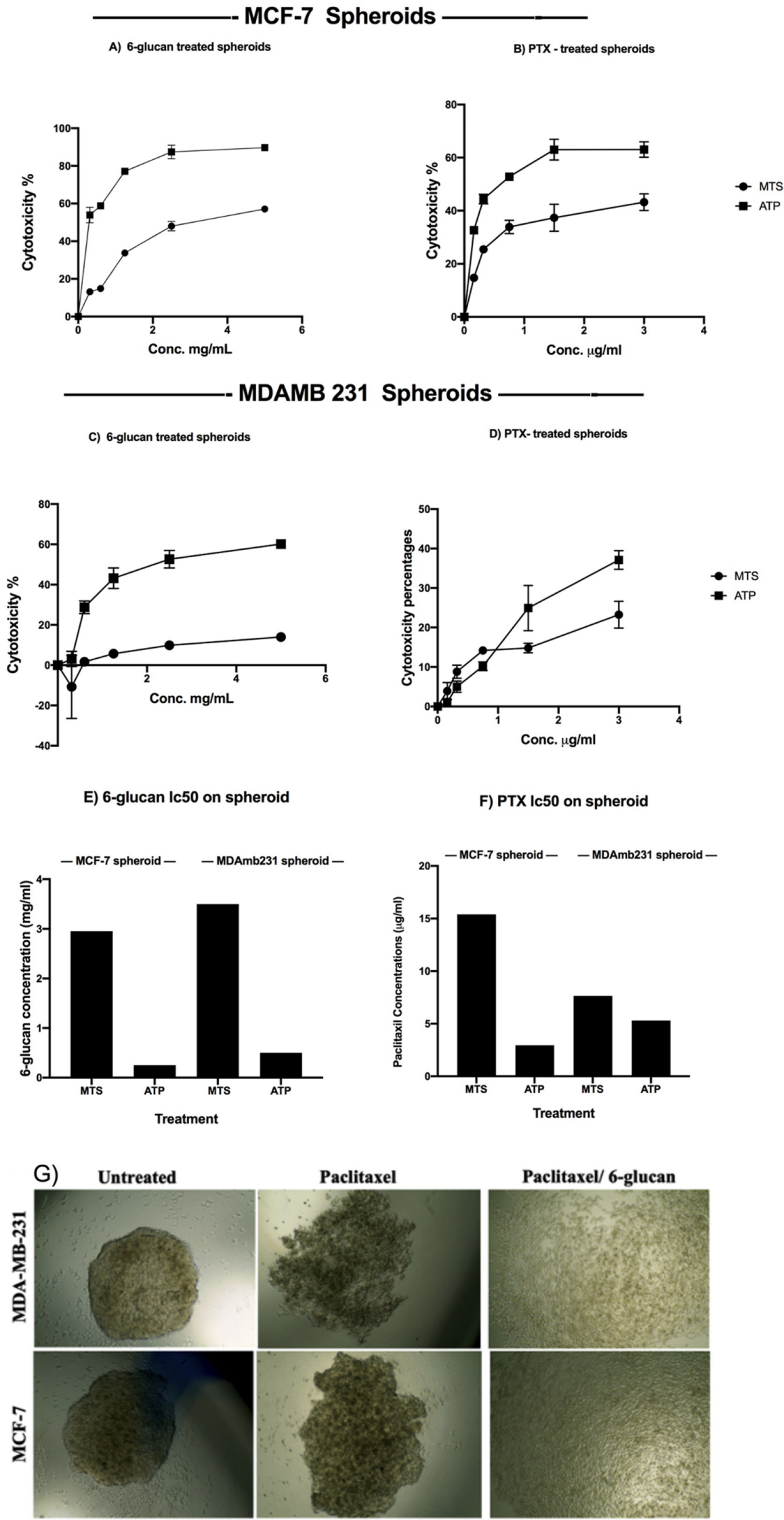
Effects of 6-glucans and PTX on mammospheres formation of the sorted CD44+/CD24−/low breast CSCs. The direct anti-tumor effect of 6-glucans and PTX were quantified against ALDH+/CD44+/CD24 − sorted MCF-7 and MDA-MB-231 cells. The formed mammospheres were treated with 6-glucans at concentrations of 0.03, 0.06, 1.25, 2.50, and 5.00, mg/ml and PTX at concentrations 0.16, 0.32, 0.75, 1.50, and 3.00 µg/ml for 24, 48, and 72 h (**A**-**D**). The IC_50_ values of 6-glucans and PTX on mammospheres of breast cancer ALDH+/CD44+/CD24 − sorted MCF-7 and MDA-MB-231 cells were calculated using GraphPad prism (**E**, **F**). The direct effects of 6-glucans and PTX on the formed mammospheres were observed under inverted microscope (**G**), 6-glucans and PTX treatments affect the ability of ALDH+/CD44+/CD24 − sorted cells to form the spheroid

In order to test the anticancer effect of 6-glucans on breast CSCs, we generated mammospheres from ALDH+/CD44+/CD24 − sorted MCF-7 and MDA-MB-231 cells (Fig. 4G) with diameters of 254.5 μm and 364.3 μm, respectively. Our results indicated that the formed mammospheres were less sensitive to 6-glucans and PTX individually. In addition, 5.0 mg/ml of 6-glucans showed superior effect over PTX until the complete inhibition of the formation of MCF-7 and MDA-MB-231 mammospheres and reduced the cellular viability with percentages of 56.52 and 12.52%, respectively (with cellular ATP reduction percentages of 90.34 and 59.33% for both mammospheres, respectively) (Fig. 4A-D). The MTS calculated IC_50_ values of 6-glucans on MCF-7 and MDA-MB-231 mammospheres were 2.9 and 3.5 mg/ml, respectively (Fig. 4E). Meanwhile, the calculated IC_50_ values of PTX on MCF-7 and MDA-MB-231 mammospheres using the MTS assay revealed 15.4 and 7.6 µg/ml, respectively (Fig. 4F). The effects of 6-glucans and PTX on cell viability and the ability of mammospheres formation were recorded after 14 days post treatment [42, 43], when 5 different doses of each treatment were used over 2 weeks (Fig. 4G). The generated mammospheres from ALDH+/CD44+/CD24 − sorted MCF-7 and MDA-MB-231 cells recorded diameters of 254.5 and 364.3 μm, respectively (Fig. 4G). It is noteworthy that the MTS calculated IC_50_ values of 6-glucans on 2-D cells didn’t differ significantly from the IC_50_ of the mammospheres, meanwhile the MTS calculated IC_50_ of PTX on MCF-7 and MDA-MB-231 mammospheres were about 5 to 100 folds higher than those obtained for the 2-D cell lines (Fig. 2B).

### Anticancer effects 6-glucans and PTX dual-therapy Cytotoxicity assay

**Fig. 5:**
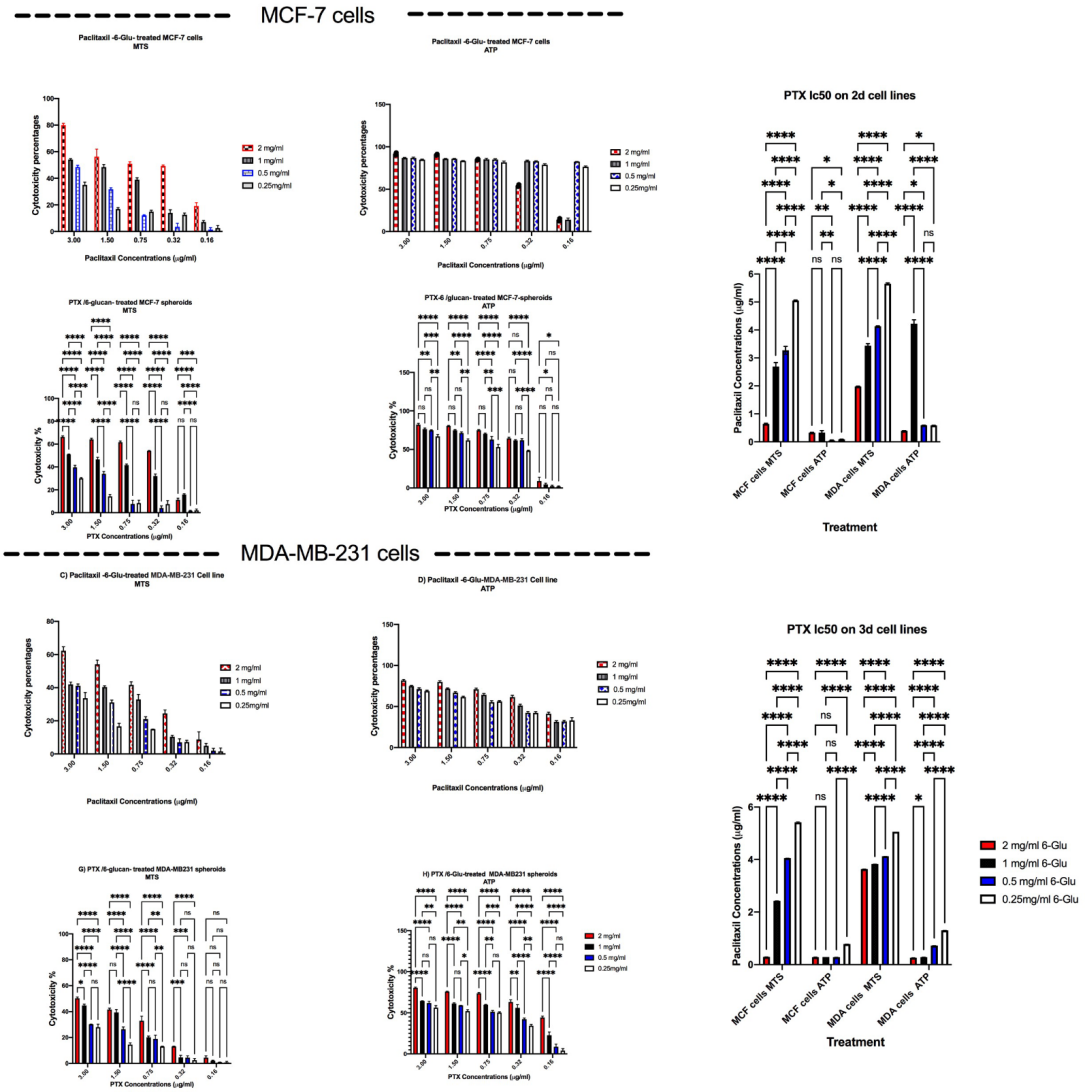
Anticancer effects and IC_50_ values of 6-glucans and PTX dual-therapy. **A**-**D** illustrate the results on MDA-MB-231 and MCF-7 2-D cell cultures. **E**-**H** illustrate the effects on 3-D cell cultures. **I**-**J** illustrate the IC50 values for PTX alone on both 2-D and 3-D cell cultures

In order to optimize the used combinatorial dose, twenty different combinations of PTX and 6-glucans were prepared by combining five concentrations of PTX (0.16, 0.32, 0.75, 1.50, and 3.00 µg/ml) with four concentrations (Fig. 5) of 6-glucans (0.25, 0.50, 1.00, and 2.00, mg/ml). After 72 h of incubation with 2-D MCF-7 and MDA-MB-231 cell lines, the IC_50_ values of PTX combined with 2.0 mg/ml 6-glucans on MCF-7 were reduced dramatically from 6.08 to 0.625 µg/ml (using MTS assay) and from 6.08 to 0.317 µg/ml upon quantifying it using ATP assay (Fig. 5). Surprisingly, 6-glucans combinations did not show any abilities to reduce the IC_50_ value of PTX on MDA-MB-231 cell line (Fig. 5). Upon testing the anticancer activities of the same combinations against MCF-7 and MDA-MB-231 mammospheres, we found a dramatically reduction in the IC_50_ values of PTX (Fig. 5). For example, the combination of 2.0 mg/ml 6-glucans with PTX significantly reduced its IC_50_ values on MCF-7 spheroids from 15.4 to 0.286 µg/ml (upon quantifying the values using MTS assay) and from 2.96 to 0.27 µg/ml (using ATP assay). However, the effect of the same combination on MDA-MB-231 mammospheres significantly reduced PTX IC_50_ from 7.64 to 3.63 µg/ml (using MTS assay) and from 5.3 to 0.255 µg/ml (using ATP assay, Fig. 5).

### Synergistic and antagonistic interactions between PTX and 6-glucans

**Fig. 6:**
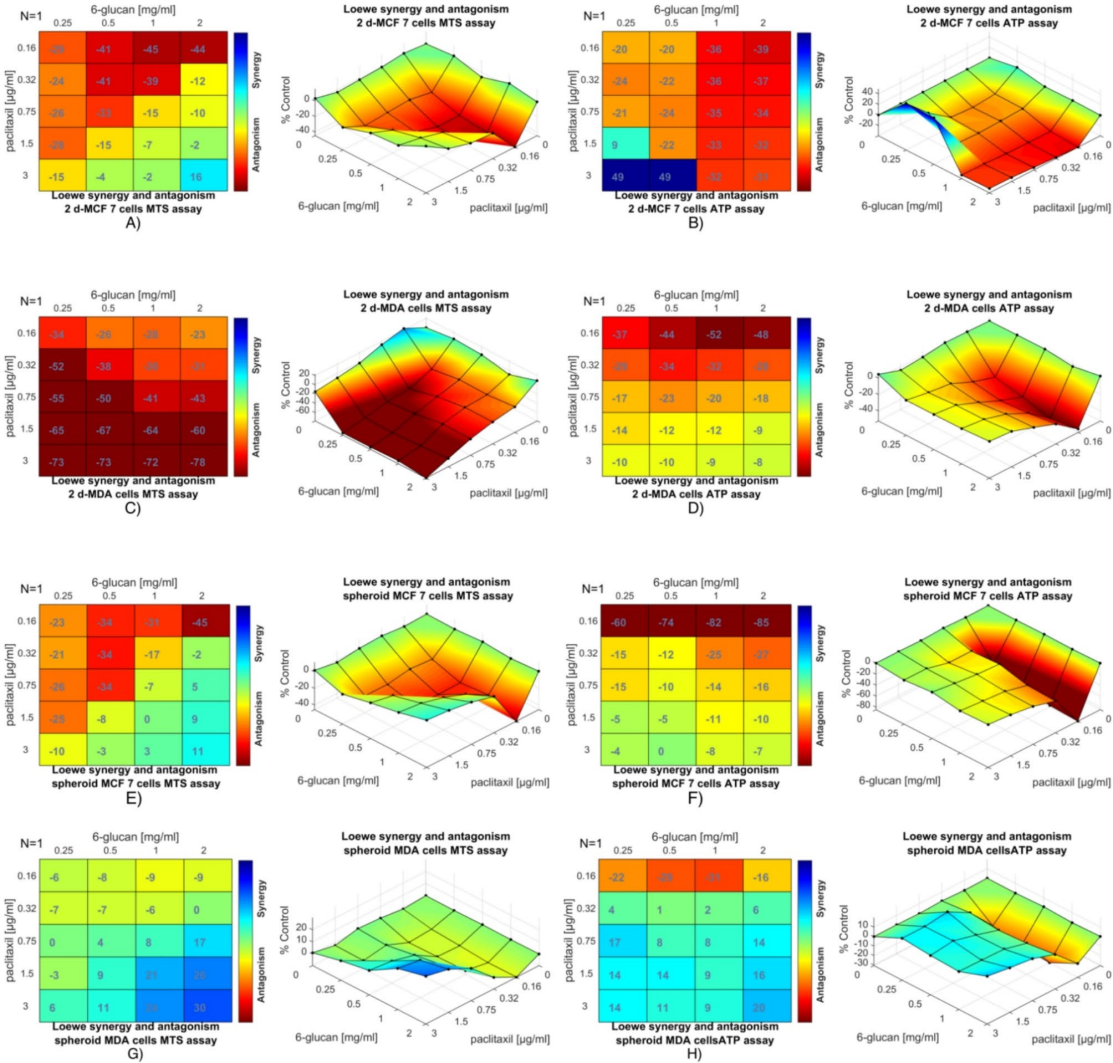
Synergistic and Antagonistic interactions between PTX and 6-glucans on breast cancer. **A**-**D** represent the results of 40 trials with varying individual and combined doses of 6-glucans (ranging from 0.25 to 2.00 mg/ml) and PTX (from 0.16 to 3.00 µg/ml). The heatmaps and 3D surface plots illustrate the extent of interaction between the drugs, indicating areas of synergy (enhanced effect) and antagonism (reduced effect) across different concentrations of PTX and 6-glucans on breast cancer viability

We used Combenefit software to perform synergism/antagonism analysis [41] and quantify the interaction level between the two drugs (6-glucan and PTX) and their combinations tested on 2D and 3D breast cancer cells using dose-response data. Combenefit calculates the difference between the Loewe model-based expected additive effect and the actual effect of the drug combination (the synergy scores). If the actual drug combination effect is greater than the additive effect, the synergy score is greater than zero and vice versa. A higher synergy score denotes greater synergy of the corresponding drug combination. As mentioned above, only the combination of 6-glucans at 2.0 mg/ml with 3.0 µg/ml PTX showed synergetic effect with a synergy score of 16 against MCF-7 cell line (which is generally more resistant than SK-BR-3 and MDA-MB-231 cell lines to cellular proliferation inhibition by individual treatment (Fig. 6). Similarly, the same combination showed a synergistic effect towards the viability of MCF-7 and MDA-MB-231 mammospheres (Fig. 6) with synergy scores of 11 and 30, respectively.

### The effect of adding 6-glucans to PTX-chemotherapy on colony formation, invasion, and migration efficacy of the CSCs sorted cells

**Fig. 7:**
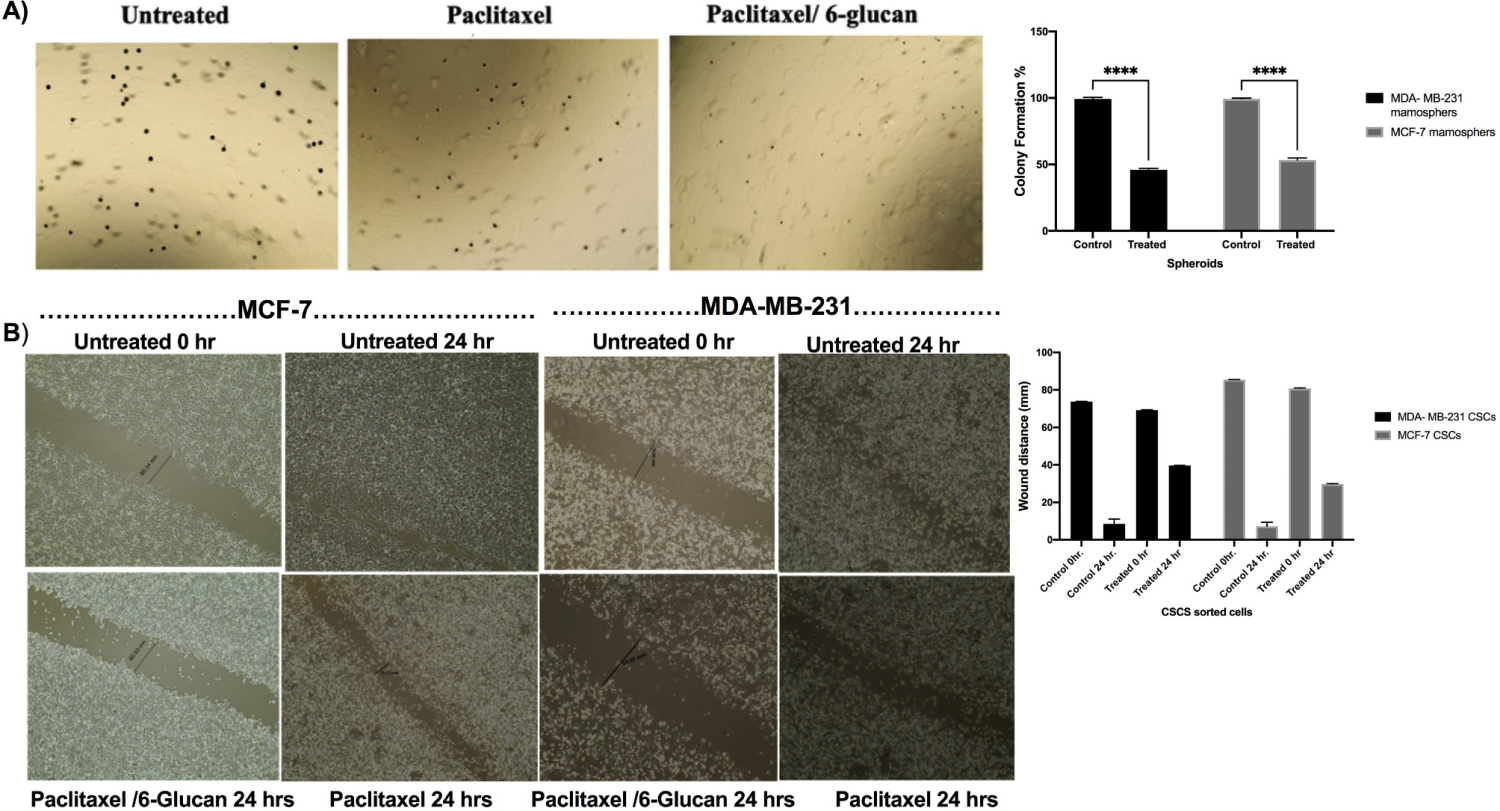
6-glucans and PTX combinations prevents migration of the CSCs sorted cells and induces apoptosis. **(A)** Colony forming assay of combination therapy. **(B)** Migration assay to quantify the effects of the selected combination in inhibiting cellular migration, the diameter of the open-wound area after 24 h of incubation was measured after and before the treatment

**Fig. 8:**
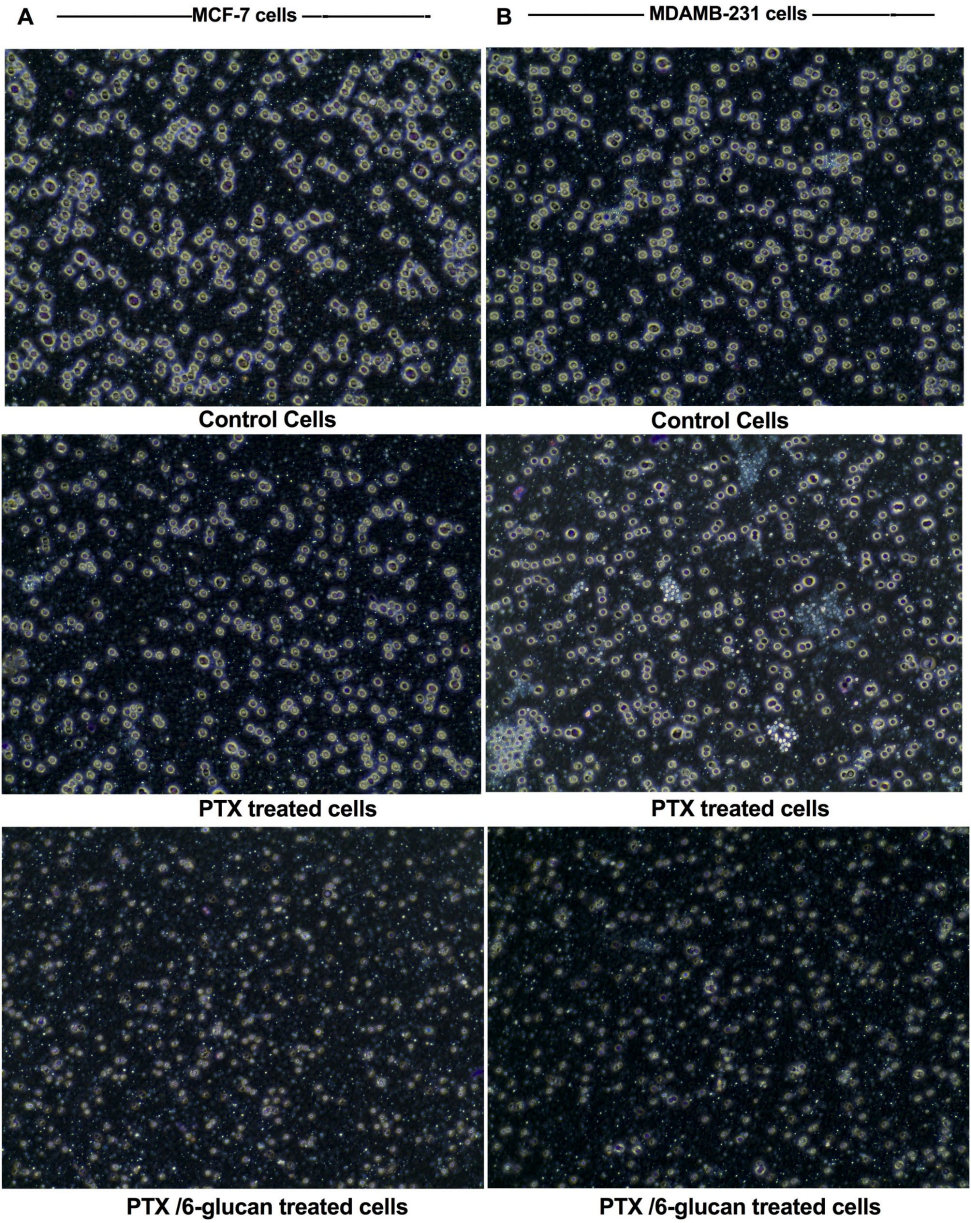
The effect of the selected combination (PTX/6 glucan) on the invasion of MCF-7 (**A**) and MDA-MB-231(**B**) mammospheres using Trans-well^®^ migration assays

The colony formation assay was utilized to evaluate the long-term survival and proliferative potential of mammospheres derived from MDA-MB-231 and MCF-7 CSCs, as depicted in Fig. 7A. The therapeutic regimen demonstrated a reduction in colony formation capabilities of both MDA-MB-231 and MCF-7 mammospheres, with a notably higher efficacy observed in MDA-MB-231 mammospheres, achieving a 57% inhibition rate as illustrated in Fig. 7A. In addition, we tested the effects of the selected combination on the invasion of MDA-MB-231 and MCF-7 mammospheres were quantified using Trans-well^®^ migration assays. Using migration assay (Fig. 7B) and Trans-well^®^ migration assay (Fig. 8), the number of migrated cells of the treated mammospheres was significantly reduced (Figs. 7B and 8) compared with the untreated cells (*p* < 0.05). Also, to quantify the effects of the selected combination on inhibiting cellular migration, the diameter of the open-wound area after 24 h of incubation was determined. Our data clearly indicated that the PTX and 6-glucans combination treatment caused a significant inhibition of cell migration after 24 h (Figs. 7B and 8).

### Adding 6-glucans to chemotherapy augments tumor cell apoptosis

**Fig. 9:**
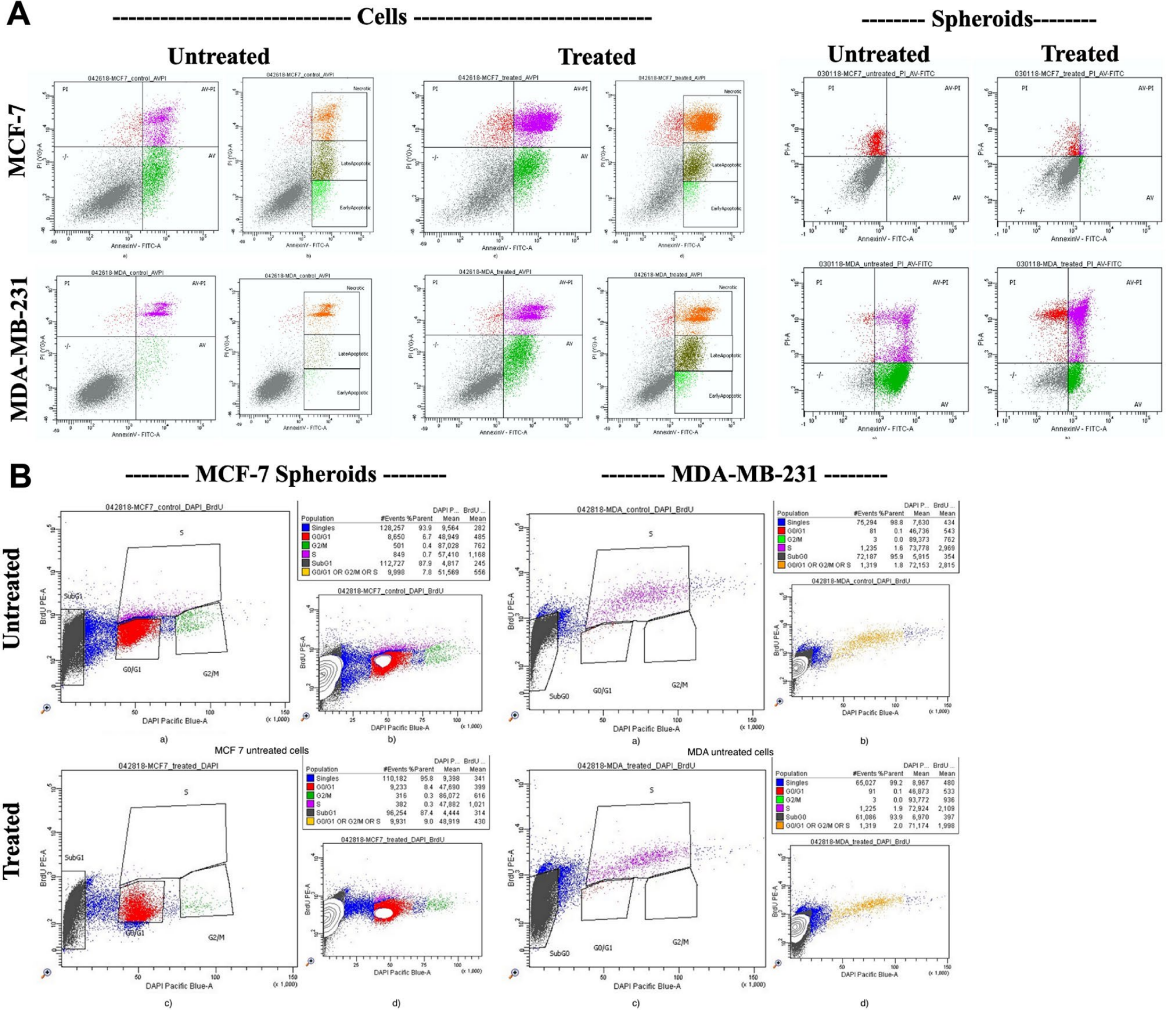
PTX and 6-glucans combination induced **A**. cancer cell apoptosis and **B**. cell cycle arrest. Annexin V-FITC/PI apoptosis detection was performed to compare control against PTX and 6-glucans combination treatment groups on both 2-D and mammospheres of MDA-MB-231 and MCF-7 CSCs sorted by flow cytometry. Cell cycle analysis of 2-D forms of MDA-MB-231 and MCF-7 CSCs cell lines were done using BrdU and flow cytometer analysis

Annexin V-FITC/PI apoptosis detection was performed to compare control against PTX and 6-glucans combination treatment groups. Both 2-D and mammospheres of MDA-MB-231 and MCF-7 CSCs sorted by flow cytometry (Fig. 9A) were used. PTX and 6-glucans combination treatment increased the apoptotic cells of MCF-7 CSCs (2-D cells) by approximately 5 folds (5.7 to 25.2%) more than the untreated cells (Fig. 9). Meanwhile, the MDA-MB-231 CSCs 2-D cells showed about 2 folds increase in the apoptotic cells after PTX and 6-glucans combination treatment (Fig. 9) more than the untreated cells (7.1 to 13.1%). Furthermore, the apoptotic cells of the treated MCF-7 mammospheres were 3 folds higher than the untreated mammospheres (from 0.7 to 2.2%). Finally, the treated MDA-MB-231 mammospheres showed higher apoptotic cells percentage than the untreated mammospheres (Fig. 9A) by about 1.5 folds (22.8 to 39%).

### Adding 6-glucans to chemotherapy induces more tumor cell cycle arrest

To understand the apoptotic mechanism induced by the developed combination therapy, we evaluated the cell cycle distribution of both 2-D and mammospheres forms of MDA-MB-231 and MCF-7 CSCs cell lines using BrdU and flow cytometer analysis. Great differences in cell cycle patterns were observed between the 2-D and mammospheres forms of CSCs. In the 2-D forms, we recorded a near normal distribution of cell cycle patterns with an increase in the percentage of cMDA-MB-231 sub G0 population from (9.4 to 26.7%) and a reduction in MCF-7 S phase population (from 31.2 to 33.9%) after treatment. While the stem cells mammospheres were mostly arrested in the sub G0/G1 phase (with over 85% of cell-arrest in the population) and remained relatively static comparing with their 2-D forms (Fig. 9B). The results also indicated that the combination treatment didn’t alter the populations in cell cycle phases significantly. Furthermore, there was about 6 folds reduction (52.4 to 9.4%) in the BrdU labeled.

### Genetic mechanism of action of combination therapy

Notably, treatment with PTX and 6-Glucan resulted in the downregulation of BCL2, Survivin, MMP7, and TGF-β in sorted MDA-MB-231 CSC mammospheres, indicating a decrease in gene expression levels associated with these cancer-related processes. In cells treated with Paclitaxel/6-glucan, all examined genes showed reduced expression levels compared to those in untreated cells. *MMP7* relative gene expression level showed 2-fold reduction in the treated cells, whereas *BCL2*, *Survivin*, and *TGF-β* showed lower relative gene expression level than the genes with 2.4, 6.2, and 1.8 × 10^− 5−^fold reduction (Fig S1), respectively. These results may explain the potential mechanism of the developed combination therapy on controlling CSCs evasion.

### PTX/6-glucan combination therapy induces apoptosis by altering mitochondrial membrane potential

**Fig. 10:**
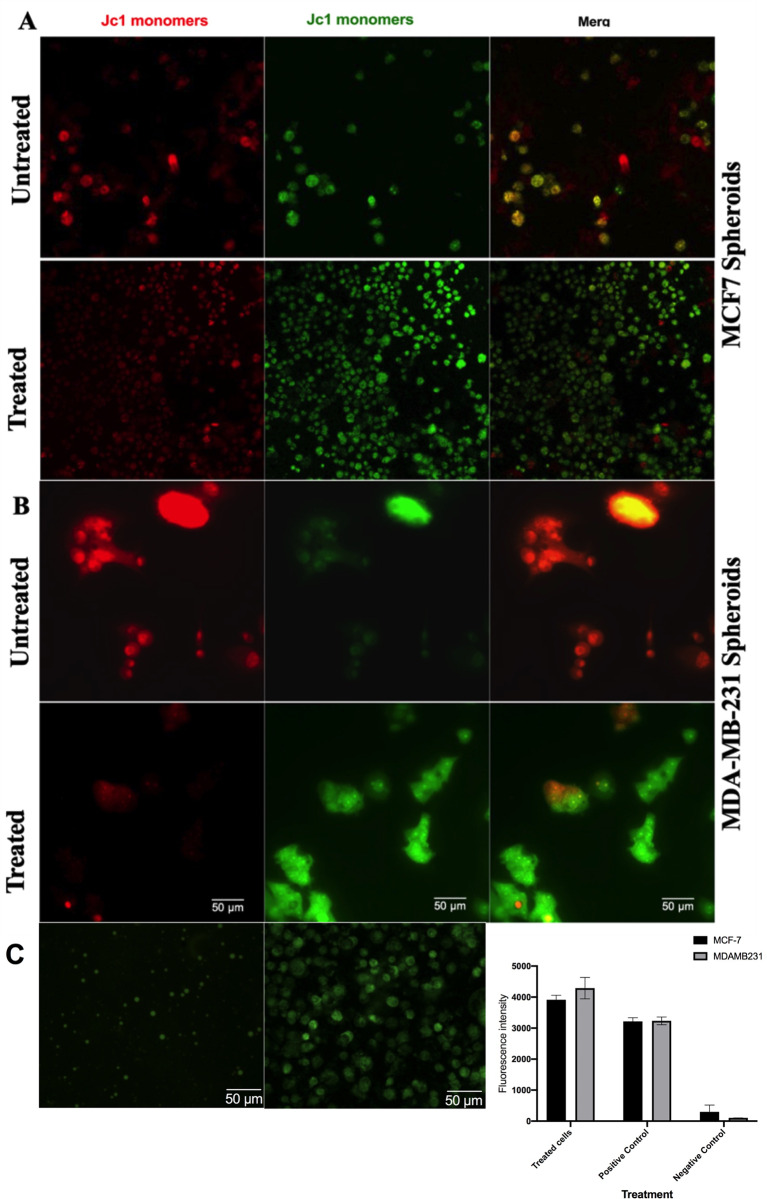
PTX and 6-glucans combination alters tumor mitochondrial membrane potential. JC-1, a dual-color probe, which selectively accumulates in viable mitochondria in a membrane potential- dependent manner, was used to assess the mitochondrial membrane potential after treatment of **(A)** MCF7 stem cells spheroids and **(B)** MDA-MB-231 stem cells spheroids. The induced ROS in spheroid cells after treatment (**C**). The induced ROS was measured (right panel) by staining the cells with DCFDA and ROS generation was observed under a fluorescence microscope at and quantified using fluorescence spectroscopy with excitation / emission at 485 nm / 535 nm. The ROS Inducer (Pyocyanin) was used as a positive control at final concentration 10 nmol

In order to test the effect of the combination therapy on CSCs mitochondrial activity, we used JC-1, which is a dual-color probe that selectively accumulates in the viable mitochondria in a membrane potential-dependent manner [44, 45]. Upon entering the cells, JC-1 accumulates inside healthy mitochondria providing high mitochondria membrane potential and forms J-aggregates with intense red fluorescence. Furthermore, in the case of dying or unhealthy cells with low mitochondrial membrane potential, JC-1 will not accumulate in the mitochondria and remains in the form of monomeric green fluorescence units. In the current study, we found that in control cells red fluorescence of J-aggregates was predominant over the green signal of J-monomers, suggesting healthy functional mitochondria with a high membrane potential. The treatment with PTX and 6-glucans reduced red fluorescence in both 2-D cells and mammospheres, and it also caused mitochondrial depolarization (Fig. 10A-B) and a small increase in intracellular ROS levels (Fig. 10C).

## Discussion

There is growing enthusiasm for the utilization of combination therapy [46–51], because of the expected greater selectivity than the traditional methods [47, 52]. Nevertheless, there are concerns that synergistic therapies would induce synergistic toxicity [53]. This could be highly expected when the mechanisms that cause side effects are closely related to those involved with therapeutic efficacy. To date, the discussion on both sides of this topic is rising. So that, our current study was designed to investigate the hypothesis that the synergistic combinations tend to be more specific and effective against breast cancer stem cells than the single drug therapy.

One of the major barriers in cancer treatment is the chemo-resistant development within the tumor microenvironment, which is known as Multi Drug Resistance (MDR) [54]. In the past, the vast majority research efforts of the chemotherapeutic resistance has focused on the cancer cells themselves, while now research reports that there are other cell types, particularly hMSCs, can act as “co-conspirators” within the tumor microenvironment, guarding cancer cells from treatment [55, 56]. These cells can be identified based on the presence of surface biomarkers such as CD44/CD24 and aldehyde dehydrogenase1 (ALDH1), in-vitro formation of spheroid or colonies, and in-vivo augmentation of tumor-initiating potential as well as tumorigenic ability [1, 57].

Targeting cancer stem-like cells is a key strategy to prevent cancer from developing drug resistance and metastasis. Therefore, we test the activity of PTX and 6-glucans individual treatment on the ALDH^+^ CD44+/CD24 − sorted MCF-7 and MDA-MB-231 mammospheres. As the use of embedded mammospheres model enables us to study the drug efficacy against the multicellular architecture. As we expected, mammospheres were less sensitive to PTX and 6-glucans treatments, independently, than the 2-D cells and the treatment completely inhibited the ability of CSCs to form mammospheres. The previously published results showed an increasing trend in cell robustness towards the tested chemotherapeutic agents, when the embedded mammospheres were tested comparing to their 2-D monolayer.

In contrast to conventional cytotoxic chemotherapy that aims to kill tumor bulk, CSC targeting therapy focuses on blocking specific signaling pathways that CSCs rely on. Thus, combining chemotherapy and CSCs targeting therapy could help in eradicating the entire tumor. Drug combination therapy can potentially reduce chemotherapy resistance and dose-based toxicity. However, interactions by the combination of drugs are likely to occur. Therefore, we select Combenefit software to detect the drug pairs that could be more effective than the individual single drugs usage. As the number of possible combinations of drugs is enormous [41], based on the analysis of mono-drug effects, we present an approach to reveal the efficacy of PTX and 6-glucans combinations against breast cancer cell lines and CSCs. These dose–response data were created during the mono-drug cytotoxicity trials and then were analyzed in terms of the presence of synergistic or antagonistic effects. Our results indicated that only the combination of 6-glucans at 2.0 mg/ml with 3.0 µg/ml PTX were found to inhibit cell proliferation in a synergetic fashion in MCF-7 cell line, out of the two tested cell lines and in both mammospheres of MDA-MB-231 and MCF-7 cell lines.

Our findings are refining the current theory, as we noticed that the mammospheres forms of CSCs are mostly arrested in the sub G0/G1 phase and are comparatively static, thus being more resistant to the chemotherapeutic drugs killing effects. Therefore, understanding the mechanism of CSCs drug resistance rather than studying the cell cycle patterns is very important for successful cancer treatment and preventing any potential recurrence [58]. In addition to constituting a major source of ATP for cancer cells, mitochondria plays an important role in controlling multiple signaling pathways, including the initiation of apoptosis by releasing Cytochrome C, responsible for the release of bioactive ROS, and the production of metabolites such as acetyl-CoA for regulating protein acetylation [59]. Hence, multiple chemotherapeutic drugs target mitochondria, either directly or through any pathway that regulate mitochondrial activity. This turns drugs into promising agents to interfere with tumor adaptations, allowing the elimination of CSCs [60]. Our results explained the ability of PTX and 6-glucans combination to induce mitochondrial depolarization in both 2-D and mammospheres. Therefore, we can show the direct effect of the selected combination on CSCs by targeting mitochondria activity and consequent ATP production.

Moreover, our findings underscore the potential of mitochondrial targeting as a therapeutic strategy against CSCs. By inducing mitochondrial depolarization with the combination of PTX and β-glucans, we effectively compromised the mitochondrial integrity crucial for CSC survival and function. This disruption not only impedes ATP production but may also interfere with mitochondrial-mediated signaling pathways that promote stemness and resistance. Targeting such fundamental aspects of CSC biology could enhance the efficacy of existing treatments and reduce the likelihood of tumor relapse.

## Electronic supplementary material

Below is the link to the electronic supplementary material.

Supplementary Material 1

## Data Availability

All data generated or analyzed during this study are included in this published article.
