# Supplementary figures and images for: Computational development of mushroom-6-glucan/paclitaxel as a synergistic complementary medicine for breast cancer therapy
Source: BMC Complement Med Ther. 2025 Feb 15;25:58. doi: 10.1186/s12906-025-04772-7 (PMC11830196; doi:10.1186/s12906-025-04772-7)

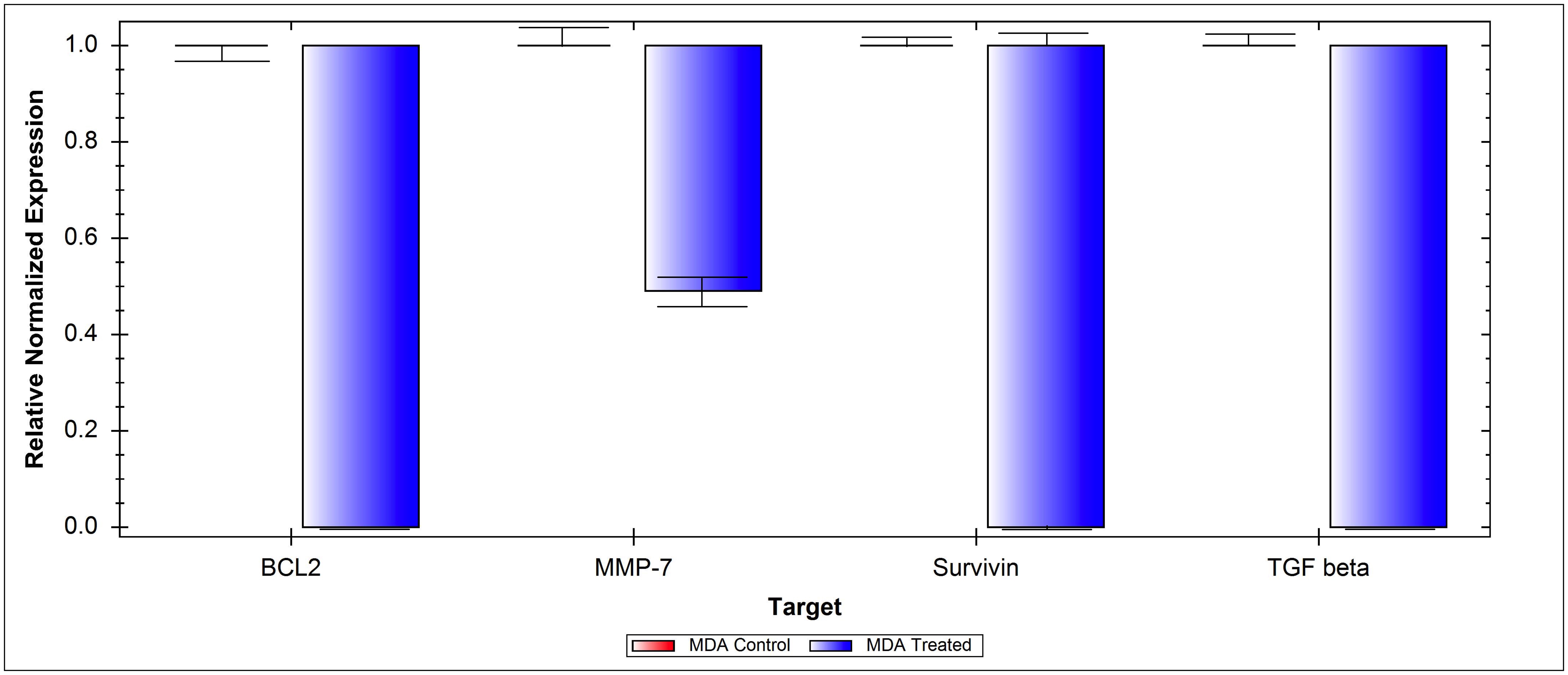

Supplement: Supplementary file 1 — Supplementary Material 1 [file 12906_2025_4772_MOESM1_ESM.jpg]
